# Ultrasound Imaging Evaluation of Textural Features in Athletes with Soleus Pathology—A Novel Case-Control Study

**DOI:** 10.3390/ijerph18041983

**Published:** 2021-02-18

**Authors:** Blanca De-la-Cruz-Torres, Emmanuel Navarro-Flores, Daniel López-López, Carlos Romero-Morales

**Affiliations:** 1 Department of Physiotherapy, University of Seville, Avicena Street, 41009 Seville, Spain; bcruz@us.es; 2 Frailty Research Organized Group (FROG), Department of Nursing, Faculty of Nursing and Podiatry, University of Valencia, 46001 Valencia, Spain; 3 Research, Health and Podiatry Group, Department of Health Sciences, Faculty of Nursing and Podiatry, Universidade da Coruña, 15403 Ferrol, Spain; daniellopez@udc.es; 4 Faculty of Sport Sciences, Universidad Europea, Villaviciosa de Odón, 28670 Madrid, Spain; carlos.romero@universidadeuropea.es

**Keywords:** soleus muscle, pathology, echotextural analysis, ultrasound imaging, athletes

## Abstract

Background: the aim of this study was to compare the echotexture of patients with soleus muscle injury and age matched controls. Methods: a sample of 62 athletes was recruited at the private clinic and was divided in two group: a healthy group (n = 31) and a soleus pathology group whose athletes had soleus muscle injury, located in the central tendon (n = 31). The muscle thickness (MTh), echointensity (EI) and echovariation (EV) were analyzed. An intra-rater reliability test (Intraclass Correlation Coefficient-ICC) was performed in order to analyze the reliability of the values of the measurements. Results: Sociodemographic variables did not show statistically significant differences (*p* > 0.05). Ultrasound imaging measurements who reported statistically significant differences were EI (*p* = 0.001) and standard deviation (SD) (*p* = 0.001). MTh and EV variables did not show statistically significant differences (*p* = 0.381 and *p* = 0.364, respectively). Moreover, reliability values for the MTh (ICC = 0.911), EI (ICC = 0.982), SD (ICC = 0.955) and EV (ICC = 0.963). Based on these results the intra-rater reliability was considered excellent. Conclusion: Athletes with a central tendon injury of soleus muscle showed a lower EI when they were compared to healthy athletes. The echogenicity showed by the quantitative ultrasound imaging measurement may be a more objective parameter for the diagnosis and follow-up the soleus muscle injuries.

## 1. Introduction

The connective tissue plays an important role within the lower limb muscles, mainly rectus femoris, biceps femoris, gastrocnemius and soleus. This structure may be named in different terms, as intramuscular tendon, connective tissue, central tendon or aponeurosis and also present variability between individuals [1].

Soleus muscle pathologies were presented in several sports modalities (e.g., soccer, basketball, volleyball, classic dance). The soleus muscle is part of the posterior region of the leg [2]. Historically, there is a lack of documentation about the morphological aspect in the dissection anatomy of the soleus muscle. Recent studies showed that soleus muscle has two aponeuroses (medial and lateral) and an intramuscular tendon (IMT). The ITM is visualized in the central part of the muscle, being an attaching point of the muscle fibers and contributing to the formation of the Achilles tendon [3,4].

Balius et al. argued that the location of five possible points in the soleus muscle where injuries may be identified: myofascial areas (anterior and posterior stress areas) and musculotendinous junction locations (proximal lateral, proximal medial and distal central tendon stress areas) [5]. In all of these places, the injury is usually more frequently located in the ITM. In this line, IMT damage is reported in the sports population, for example in the rectus femoris and soleus muscle injuries. The IMTs feature a high stiffness; intramuscular tendon ruptures that may cause the formation of hypertrophic intramuscular connective tissue scar into the tendon; moreover, the sensation of a progressive tightness during activity or lack of flexibility as the main symptoms [6].

Recent research suggests that sometimes soleus injuries do not have a proper diagnosis which has consequences on its evolution, increasing the athletes’ risk of reinjury [7,8]. Several authors suggested that ultrasound (US) was not a sensitive method for the detection and evaluation of soleus muscle disturbances with respect to magnetic resonance imaging (MRI), however it is possible to increase this sensitivity if the therapists have proper anatomic knowledge and ultrasound examination procedures [9]. However, in 2017, Loizides et al. suggested that US may be a useful tool to help the clinician in assessing the main cause of muscle injury [10].

Ultrasonography is an accessible, noninvasive and economical tool that allows us to obtain images for the diagnosis of musculoskeletal injuries by clinicians [11]. Echogenicity, based on the pixel intensity quantification of an ultrasound image, could express different physiological views according to the literature and their study and utilization has increased in the last few years in order to provide a better understanding of ultrasound imaging analysis. Echointensity (EI) has been applied to the analysis of muscle strength in middle-aged and older adult populations [12], evaluating the muscle response to some types of exercise [13,14] or quantifying muscle size [15,16]. Moreover, EI has been considered a power biomarker for identifying the disruption of muscle structure in some degenerative muscle pathologies, for example lateral amyotrophic sclerosis [17,18] or muscle sarcopenia [19]. EI modifications have been observed as the disruption of normal connective tissue muscle in both acute [20] and degenerative conditions [21,22]. However, the role of the EI parameter as diagnostic or follow-up biomarkers in musculoskeletal damage is limited. Finding a muscle ultrasonography biomarker not influenced by factors (e.g., pain, athletes feeling, functional test…) factors other than pathology will be a great advance. Sometimes, clinicians may think that an injury is healed when a correct ultrasound image is correlated with good sensations and good functional tests of the athletes. However, it is important for clinicians to ensure that the tissue is completely healed to prevent or avoid re-injury. In this line, echovariation (EV) shows the degree of gray dispersion from the average value determined by the relationship between the standard deviation (SD) and the mean pixel value of the histograms obtained from the range of interest (ROI). [17,23] EV, in combination with EI, tries to improve the interpretation of the muscle echogenicity. To our knowledge, EV has been studied to characterize the plantar fascia in asymptomatic and symptomatic subjects [24], multifidus and thoracolumbar fasciae in subjects with and without chronic lumbopelvic pain [25], Achilles tendon in asymptomatic dancers [26] and patients with amyotrophic lateral sclerosis [23].

We hypothesize that the athletes with soleus pathology, located in the central tendon (CT), may show differences in soleus echotexture (EI and EV) variables with respect to healthy athletes because of the appearance of disrupted intra-muscular structure [6]. Therefore, the aim of this study was to compare the echotexture of patients with soleus muscle injury, located in the CT and age matched controls.

## 2. Materials and Methods

### 2.1. Design

A prospective, cross-sectional, case-control study was carried out in an athletic population according to the Strengthening the Reporting of Observational Studies in Epidemiology Statement (STROBE) recommendations.

### 2.2. Ethical Considerations

The Declaration of Helsinki was respected throughout the study and also the study was approved by the Ethics Committee of University Hospital Virgen Macarena-Virgen del Rocio (Reference code: 06/2019). In addition, all the individuals signed written informed consent form before participating in this study.

### 2.3. Sample Size Calculation

The sample size calculation was carried out with G*Power software by the difference between healthy group and patient group using the EI variable of a pilot study (n = 14) divided into two groups (mean ± SD), 7 subjects with soleus pathology (49.43 ± 13.59) and 7 individuals for the healthy group (59.55 ± 17.37). An α error of 0.05, a power of 0.80 and effect size of 0.64 with 1 tailed hypothesis were used for the sample size calculation. Finally, a total sample of 62 subjects was calculated.

### 2.4. Participants

A total sample of 62 subjects were recruited and divided based on the echogenicity of the soleus muscle in both groups. Precisely, a soleus muscle injury, located in the central tendon, was considered in the present study, according to the following parameters: had symptoms for at least six months; and a self-rated progressive tightness score greater than or equal to 4 cm during sport activities or during the soleus stretching, measured using the numeric rate scale (NRS) [27]. The recruiting process was developed by a private clinic specializing in musculoskeletal injuries and sport medicine. Before the physical examination, a clinical history was taken. The presence of a central intramuscular tendon injury of the soleus muscle was assessed based on a clinical examination and clinical history of the participants by an expert therapist and confirmed using US examination performed by a qualified clinician with more than 15 years of experience in ultrasound imaging.

The selection criteria for the patients were (a) age greater than 18 years old; (b) athletes who participated in sports that involve lower limbs; (b) at least of five-years practicing the sport; (c) training at least two hours, four days a week; (d) non-acute lower limb pathology at least six months ago; (e) non low back musculoskeletal disorders. Furthermore, inclusion criteria to be included for the healthy group was being a healthy athlete; and for the pathological group, having a clinical diagnosis of soleus muscle injury, characterized by muscle stiffness and lack of elasticity when stretching the muscle and corroborated by an experienced clinician.

### 2.5. Clinical Variables

Clinical and demographic variables were recorded, such as age, height, weight, body mass index (BMI), sex, dominance side, injured side, sport training (years) and average sport hours per week. Severity of progressive tightness during sport activities or during the soleus stretching and pain level at palpation in the injured location was evaluated using NRS (0 point, no symptoms; 10 points, worse symptoms).

### 2.6. Ultrasonography

**Figure 1:**
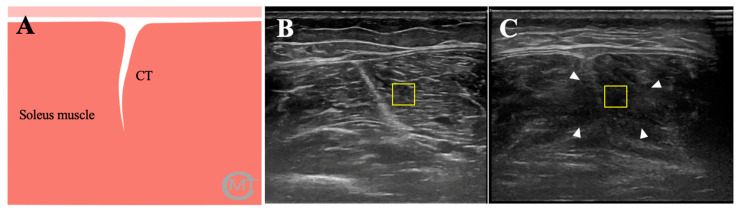
Ultrasound images of central tendon of soleus muscle. (**A**). Graphic representation of the ultrasound image of the soleus muscle; (**B**). Ultrasound imaging of health athlete; (**C**). Ultrasound imaging of an athlete with soleus muscle injury. Ultrasound imaging of patients group shows muscle fiber disorganization at the level of tear (arrowhead). CT, central tendon; ROI.

All US imaging evaluation was carried out by an experienced therapist, who was blind to the subjects’ muscle status, with more than 15 years of experience in US imaging. A high-definition US with a 6-15 MHz linear transducer (S7, General Electrical Healthcare, USA) was employed to perform all US images. For the classification into either the healthy or pathology group, US examination was carried out in a prone position with the feet hanging over the end of the table. In addition, the transducer was located in the transverse plane at the central tendon in order to describe the echogenic pattern (Figure 1). US images were recorded and stored with a depth of 4 cm, 12 MHz of frequency, 51 points of gain, neutral position of the time gain compensation, 2 focus located at 1 and 2 cm each, respectively, in the CT of the soleus muscle for keeping constant throughout the study. To reduce the presence of artifacts, the angle of the probe was adjusted until the best muscle EI was obtained in each image. Three images were captured with an imaging resolution of 820 × 614 pixels with 256 gray-scale levels and were saved as. TIFF files without compression or losses.

### 2.7. Image Analysis

Quantitative US variables, including thickness, EI and EV were taken in both groups. The muscle thickness (MTh) was described as the distance between superficial and deep fascia of the muscle. The imaging analysis was developed offline with the ImageJ software (Bethesda, Maryland, USA) by a blinded examiner. Moreover, according to research by Fukumoto et al. [12], an ROI of 64 × 64 with an 8-bit gray scale using the ROI Manager tool was executed to extract the pixel distribution histogram. The ROI was described as the muscle area without bone and fascia with the best reflection (Figure 1). Subsequently, echotexture values were obtained from the histogram where EI was defined as the mean value of the gray-scale distribution of the pixels and EV was described as the relation between SD and the mean of pixel distribution (EV = σ/μ × 100), where σ is the SD of the image intensities and μ is the mean value of intensity in each ROI of the three images previously acquired.

The ROIs’ size were the same in all subjects and the position was the one that best represented the characteristics of the muscle. According to Caresio et al., the authors knew that the muscle echo-intensity depends on different factors such as ROI size, shape and location [28]. To minimize these possible mistakes, the intra-observer reliability was established on a set of 15 images as sufficient for clinical measurement.

### 2.8. Statistics Analysis

The statistical analysis was performed using the SPSS package v.22.0 (IBM, Armonk, NY: IBM Corp). First, Kolmogorov-Smirnov was performed to check the normality assumption. Second, a descriptive analysis was employed for all the individuals and separately in the two groups. Finally, a comparative analysis between healthy group and patient group was developed. Mean, SD with the Student-s *t* test and median, interquartile range (IR) with Mann-Whitney *U* test were carried out for parametric and non-parametric data, respectively. In addition, Levene’s test was employed to assess the equality of variances. Moreover, the intraclass correlation coefficient (ICC) was performed to evaluate the intra-rater reliability of all the ultrasound measurements.

## 3. Results

Considering the Table 1, sociodemographic variables did not show statistically significant differences (*p* > 0.05). Ultrasound imaging measurements who reported statistically significant differences were EI (*p* = 0.001) and SD (*p* = 0.001). MTh and EV variables did not show statistically significant differences (*p* = 0.381 and *p* = 0.364, respectively). Moreover, reliability values for the MTh (ICC = 0.911), EI (ICC = 0.982), SD (ICC = 0.955) and EV (ICC = 0.963). Based on these results, the intra-rater reliability was considered excellent.

## 4. Discussion

In the present cross-sectional study, the assessment of the echoestructure and echotexture, as well as the intra-rater reliability, of each variable was performed in the soleus muscle of athletes with and without pathology. The main finding of the present study was that echotexture features differentiated athletes with a CT injury of soleus muscle from the healthy athletes. Specifically, the results showed that a lower EI in the pathology group compared with the healthy group and also no differences were observed regarding MTh and EV. In addition, an intra-rater reliability test was performed with the aim of increasing the reliability of the values of the measurements. Our scores showed a high reliability in all variables of the present study for this sample.

Results reported in the present study are in line with previous studies which analyze the EI parameter in different muscle conditions. EI of the selected regions has been considered a useful biomarker reflecting musculoskeletal quality and disturbances related to pathological conditions. Heckmatt et al. [28] compared the EI of healthy and muscle dystrophy individuals and reported changes due to the fat infiltration of connective tissue layers into the muscle [29]. Thus, the authors suggested that the EI variable was adequate for the description of the muscle structure in order to assess muscle quality in different muscle situations. In addition, several authors reported that an increase of EI in degenerative alterations could be related to a fat and collagen infiltration [21,22]. Contrarily, this study showed a minor EI in athletes with soleus injury, which meant a lack of structure and less homogeneity in muscle, characterized by the presence of a hypoechoic pattern, is considered to have a lower echogenicity. Similar data of lower values were found in other studies where EI was evaluated in different tendons [26,30]. To our knowledge, there are limited studies reporting on the changes to EI in muscle injuries in the sports field and this is the first study to apply an EI analysis to the soleus muscle injury in athletes.

Currently, there is a high interest in studying soleus muscle characteristics due to its importance during sporting activities as well as for a better understanding of its pathological features [5,6,31,32]. A soleus muscle pathology may be misdiagnosed and underestimated because of its special clinical features and that, in many cases, soleus disturbances do not require athletes to stop training or playing games [6]. The soleus tears diagnosis is often incorrect due to clinicians considering that US is not a sensitive tool for detecting soleus injuries with respect to MRI [9], although if clinicians performed an anatomically-based ultrasound examination, this sensitivity may be enhanced. According to the results of this study, the authors suggested that the use of echotextural parameters, such as EI, may be used as a complementary test for diagnosis and follow-up in the management of soleus injuries.

Regarding the EV and MTh parameters, this study did not show differences between groups. Several authors hypothesized the possibility of developing a muscle biomarker with ultrasonography, for example, Martínez-Payá et al. found a decrease in the EV of the quadriceps femoris, tibialis anterior, biceps brachialis and forearm flexor group in patients with amyotrophic lateral sclerosis. Almazán-Polo et al. [17] observed that in EV of the lumbar muscle in athletes with and without chronic lumbopelvic pain no differences were found [25]. To date, the limited studies on the evaluation of the behavior of EV in muscles limits the opportunity to draw overly categorical conclusions from our results. The authors suggest that the knowledge of the tissue characteristics of ITM tears, especially of soleus injury, is still limited. In fact, there could be hypoechoic regions next to fibrotic regions that may affect the results. Thus, further studies are needed in order to improve the interpretation of this variable as a potential biomarker in musculoskeletal structures. Regarding MTh, the scientific literature shows that there are differences between patients and heathy subjects. For example, Martínez-Payá et al. found that amyotrophic lateral sclerosis patients presented a minor thickness of several muscle than healthy subjects. Calvo-Lobo et al. [17] showed that athletes with lumbo-pelvic pain had a reduced diaphragm thickness compared to healthy athletes [33]. In the case of our study, no differences were observed comparing both groups. The authors hypothesize that this soleus tear is a pathological condition that does not force the athlete to stop and, therefore, MThs were not affected. In this line, further studies are also necessary to better understand the effect of the pathological conditions on the MTh.

### 4.1. Clinical Implications

The most important clinical application of the present study highlighted the benefits of the EI as a more objective guideline for the classification of the echogenic pattern applied to the muscle. In addition, EI may be used in future studies for the management and follow up of musculoskeletal disturbances, as well as in preventive and management exercise programs.

### 4.2. Limitations and Future Lines

Several limitations were observed for the present study. Despite the excellent intra-rater results for the reliability, no inter-rater procedure was developed and future inter-rater reliability studies are still needed in order to standardize protocols and methods for quantitative ultrasound examinations in muscle and soft tissues. Thus, further research could be considered in musculoskeletal disturbances and other areas. Moreover, future studies are needed for EI and EV variables as descriptors of the soft tissues features.

## 5. Conclusions

The use of this new echotexture parameters measured with ultrasound imaging is a promising biomarker in musculoskeletal disorders. The authors suggest that EI could be employed to differentiate athletes with soleus muscle pathology from healthy athletes due to soleus tear being characterized by a minor EI. The echogenicity reported by the quantitative US measurement may be an objective parameter for the diagnosis, management and follow-up of soleus muscle injuries.

## Figures and Tables

**Table 1:** Sociodemographic data, pain scores and echotexture variables of the sample.

Data	Total (n = 62)	Healthy Group (n = 31)	Patient Group (n= 31)	*p*-Value
Age, y	27.00 ± 13.50 †	27.00 ± 16.00 †	27.00 ± 12.00 †	0.994 ‡
Weight, kg	65.75 ± 16.65 †	66.00 ± 19.00 †	65.00 ± 16.00 †	0.390 ‡
Height, m	1.69 ± 0.15 *	1.70 ± 0.09 *	1.67 ± 0.08 *	0.095 **
BMI, kg/m^2^	23.26 ± 3.05 †	23.41 ± 2.89 †	23.18 ± 3.01 †	0.938 ‡
NRS	5.03 ± 1.38 †	n/a	5.03 ± 1.38 †	n/a
NRS at palpation	5.81 ± 1.53 †	n/a	5.81 ± 1.53 †	n/a
MTh (mm)	17.94 ± 2.31 *	17.68 ± 1.77 *	18.20 ± 2.75 *	0.381 **
Echointensity (EI)	50.17 ± 18.94 *	63.74 ± 12.86 *	36.71 ± 13.78 *	0.001 **
Echovariation (EV)	30.93 ± 12.11 †	33.07 ± 10.13 †	28.69 ± 16.75 †	0.364 ‡

Abbreviations: NRS, numeric rate scale; MTh, muscle thickness. * Mean ± standard deviation (SD) was applied. ** Student´s *t*-test for independent samples was performed. † Median ± interquartile range (IR) was used. ‡ Mann-Whitney *U* test was utilized.

## Data Availability

The data presented in this study are available on request from the corresponding author. The data are not publicly available due to ethical condition.

## References

[1] Brukner P.D., Connell D. (2015). ‘Serious thigh muscle strains’: Beware the intramuscular tendon which plays an important role in difficult hamstring and quadriceps muscle strains. Br. J. Sports Med..

[2] Koulouris G., Ting A.Y.I., Jhamb A., Connell D., Kavanagh E.C. (2007). Magnetic resonance imaging findings of injuries to the calf muscle complex. Skelet. Radiol..

[3] Courthaliac C., Weilbacher H. (2007). Imaging of painful calf in athletes. J. Radiol..

[4] Śmigielski R. (2008). Management of Partial Tears of the Gastro-Soleus Complex. Clin. Sports Med..

[5] Balius R., AlOmar X., Rodas G., Miguel-Pérez M., Pedret C., Dobado M.C., Blasi J., Koulouris G. (2012). The soleus muscle: MRI, anatomic and histologic findings in cadavers with clinical correlation of strain injury distribution. Skelet. Radiol..

[6] Brukner P., Cook J.L., Purdam C.R. (2018). Does the intramuscular tendon act like a free tendon?. Br. J. Sports Med..

[7] Orchard J., Best T.M. (2002). The Management of Muscle Strain Injuries: An Early Return Versus the Risk of Recurrence. Clin. J. Sport Med..

[8] Orchard J.W., Best T.M., Mueller-Wohlfahrt H.-W., Hunter G., Hamilton B.H., Webborn A., Jaques R., Kenneally D., Budgett R., Phillips N. (2008). The early management of muscle strains in the elite athlete: Best practice in a world with a limited evidence basis. Br. J. Sports Med..

[9] Balius R., Rodas G., Pedret C., Capdevila L., AlOmar X., Bong D.A. (2014). Soleus muscle injury: Sensitivity of ultrasound patterns. Skelet. Radiol..

[10] Loizides A., Gruber H., Peer S., Plaikner M. (2017). Muscular injuries of athletes: Importance of ultrasound. Radiologe.

[11] Åström M., Gentz C.-F., Nilsson P., Rausing A., Sjöberg S., Westlin N. (1996). Imaging in chronic achilles tendinopathy: A comparison of ultrasonography, magnetic resonance imaging and surgical findings in 27 histologically verified cases. Skelet. Radiol..

[12] Fukumoto Y., Ikezoe T., Yamada Y., Tsukagoshi R., Nakamura M., Mori N., Kimura M., Ichihashi N. (2012). Skeletal muscle quality assessed from echo intensity is associated with muscle strength of middle-aged and elderly persons. Graefe’s Arch. Clin. Exp. Ophthalmol..

[13] Wong V., Abe T., Chatakondi R.N., Bell Z.W., Spitz R.W., Dankel S.J., Loenneke J.P. (2019). The influence of biological sex and cuff width on muscle swelling, echo intensity, and the fatigue response to blood flow restricted exercise. J. Sports Sci..

[14] Wong V., Spitz R.W., Bell Z.W., Viana R.B., Chatakondi R.N., Abe T., Loenneke J.P. (2020). Exercise induced changes in echo intensity within the muscle: A brief review. J. Ultrasound.

[15] Medeiros D.M., Mantovani R.F., Lima C.S. (2017). Effects of low-intensity pulsed ultrasound on muscle thickness and echo intensity of the elbow flexors following exercise-induced muscle damage. Sport Sci. Health.

[16] Yoshiko A., Tomita A., Ando R., Ogawa M., Kondo S., Saito A., Tanaka N.I., Koike T., Oshida Y., Akima H. (2018). Effects of 10-week walking and walking with home-based resistance training on muscle quality, muscle size, and physical functional tests in healthy older individuals. Eur. Rev. Aging Phys. Act..

[17] Martínez-Payá J.J., Ríos-Díaz J., Del Baño-Aledo M.E., Tembl-Ferrairó J.I., Vazquez-Costa J.F., Medina-Mirapeix F. (2017). Quantitative Muscle Ultrasonography Using Textural Analysis in Amyotrophic Lateral Sclerosis. Ultrason. Imaging.

[18] Arts I.M., Overeem S., Pillen S., Kleine B.U., Boekestein W.A., Zwarts M.J., Schelhaas H.J. (2012). Muscle ultrasonography: A diagnostic tool for amyotrophic lateral sclerosis. Clin. Neurophysiol..

[19] Ticinesi A., Meschi T., Narici M.V., Lauretani F., Maggio M. (2017). Muscle Ultrasound and Sarcopenia in Older Individuals: A Clinical Perspective. J. Am. Med Dir. Assoc..

[20] Yitzchaki N., Zhu W.G., Kuehne T.E., Vasenina E., Dankel S.J., Buckner S.L. (2020). An examination of changes in skeletal muscle thickness, echo intensity, strength and soreness following resistance exercise. Clin. Physiol. Funct. Imaging.

[21] Pillen S., Tak R.O., Zwarts M.J., Lammens M.M., Verrijp K.N., Arts I.M., Van Der Laak J.A., Hoogerbrugge P.M., Van Engelen B.G., Verrips A. (2009). Skeletal Muscle Ultrasound: Correlation Between Fibrous Tissue and Echo Intensity. Ultrasound Med. Biol..

[22] Martínez-Payá J.J., Ríos-Díaz J., Medina-Mirapeix F., Vázquez-Costa J.F., Del Baño-Aledo M.E. (2018). Monitoring Progression of Amyotrophic Lateral Sclerosis Using Ultrasound Morpho-Textural Muscle Biomarkers: A Pilot Study. Ultrasound. Med. Biol..

[23] Martínez-Payá J.J., Del Baño-Aledo M.E., Ríos-Díaz J., Tembl-Ferrairó J.I., Vázquez-Costa J.F., Medina-Mirapeix F. (2017). Muscular Echovariation: A New Biomarker in Amyotrophic Lateral Sclerosis. Ultrasound Med. Biol..

[24] Ríos-Díaz J., Martínez-Payá J.J., Del Baño-Aledo M.E., De Groot-Ferrando A., Botía-Castillo P., Fernández-Rodríguez D. (2015). Sonoelastography of Plantar Fascia: Reproducibility and Pattern Description in Healthy Subjects and Symptomatic Subjects. Ultrasound Med. Biol..

[25] Almazán-Polo J., López-López D., Romero-Morales C., Rodríguez-Sanz D., Becerro-De-Bengoa-Vallejo R., Losa-Iglesias M.E., Bravo-Aguilar M., Calvo-Lobo C. (2020). Quantitative Ultrasound Imaging Differences in Multifidus and Thoracolumbar Fasciae between Athletes with and without Chronic Lumbopelvic Pain: A Case-Control Study. J. Clin. Med..

[26] De-La-Cruz-Torres B., Barrera-García-Martín I., Almazán-Polo J., Jaén-Crespo G., Romero-Morales C. (2020). Ultrasound imaging evaluation of structural and textural features in asymptomatic achilles tendons in pre-professional dancers: A cross-sectional study. Phys. Ther. Sport.

[27] Downie W.W., Leatham P.A., Rhind V.M., Wright V., Branco J.A., Anderson J.A. (1978). Studies with pain rating scales. Ann. Rheum. Dis..

[28] Caresio C., Molinari F., Emanuel G., Minetto M.A. (2014). Muscle echo intensity: Reliability and conditioning factors. Clin. Physiol. Funct. Imaging.

[29] Heckmatt J., Dubowitz V., Leeman S. (1980). Detection of pathological change in dystrophic muscle with B-scan ultrasound imaging. Lancet.

[30] Giacchino M., Caresio C., Gorji N., Molinari F., Massazza G., Minetto M. (2019). Quantitative analysis of patellar tendon size and structure in asymptomatic professional players: Sonographic study. Muscle Ligaments Tendons J..

[31] Romero-Morales C., Calvo-Lobo C., Navarro-Flores E., Mazoteras-Pardo V., García-Bermejo P., López-López D., Martínez-Jiménez E.M., De-La-Cruz-Torres B. (2020). M-mode Ultrasound Examination of Soleus Muscle in Healthy Subjects: Intra- and Inter-Rater Reliability Study. Healthcare.

[32] De-La-Cruz-Torres B., Barrera-García-Martín I., Valera-Garrido F., Minaya-Muñoz F., Romero-Morales C. (2020). Ultrasound-Guided Percutaneous Needle Electrolysis in Dancers with Chronic Soleus Injury: A Randomized Clinical Trial. Evidence-Based Complement. Altern. Med..

[33] Calvo-Lobo C., Almazán-Polo J., Becerro-De-Bengoa-Vallejo R., Losa-Iglesias M.E., Palomo-López P., Rodríguez-Sanz D., López-López D. (2019). Ultrasonography comparison of diaphragm thickness and excursion between athletes with and without lumbopelvic pain. Phys. Ther. Sport Off. J. Assoc. Chart. Physiother. Sports Med..

